# First Case Report of Acute Renal Failure After Mesh-Plug Inguinal Hernia Repair in a Kidney Transplant Recipient

**DOI:** 10.1097/MD.0000000000003199

**Published:** 2016-04-01

**Authors:** Massimiliano Veroux, Vincenzo Ardita, Domenico Zerbo, Pietro Caglià, Stefano Palmucci, Nunziata Sinagra, Alessia Giaquinta, Pierfrancesco Veroux

**Affiliations:** From the Vascular Surgery and Organ Transplant Unit (MV, VA, DZ, PC, NS, AG, PV), Department of Medical and Surgical Sciences and Advanced Technologies; and Radiodiagnostic and Radiotherapy Unit (SP), University Hospital of Catania, Italy.

## Abstract

Acute renal failure due to ureter compression after a mesh-plug inguinal repair in a kidney transplant recipient has not been previously reported to our knowledge. A 62-year-old man, who successfully underwent kidney transplantation from a deceased donor 6 years earlier, was admitted for elective repair of a direct inguinal hernia. The patient underwent an open mesh-plug repair of the inguinal hernia with placement of a plug in the preperitoneal space. We did not observe the transplanted ureter and bladder during dissection of the inguinal canal. Immediately after surgery, the patient became anuric, and a graft sonography demonstrated massive hydronephrosis. The serum creatinine level increased rapidly, and the patient underwent an emergency reoperation 8 hours later. During surgery, we did not identify the ureter but, immediately after plug removal, urine output increased progressively. We completed the hernia repair using the standard technique, without plug interposition, and the postoperative course was uneventful with complete resolution of graft dysfunction 3 days later.

Furthermore, we reviewed the clinical features of complications related to inguinal hernia surgery. An increased risk of urological complications was reported recently in patients with a previous prosthetic hernia repair undergoing kidney transplantation, mainly due to the mesh adhesion to surrounding structures, making the extraperitoneal dissection during the transplant surgery very challenging. Moreover, older male kidney transplant recipients undergoing an inguinal hernia repair may be at higher risk of graft dysfunction due to inguinal herniation of a transplanted ureter.

Mesh-plug inguinal hernia repair is a safe surgical technique, but this unique case suggests that kidney transplant recipients with inguinal hernia may be at higher risk of serious urological complications. Surgeons must be aware of the graft and ureter position before proceeding with hernia repair. A prompt diagnosis with graft sonography and abdominal computed tomography scan and emergency surgery may avoid the need for nephrostomy and may resolve graft dysfunction more rapidly.

## INTRODUCTION

Kidney transplantation is the optimal replacement therapy for patients with end-stage renal disease. Impressive improvements in surgical techniques and the routine use of intraoperative ureteric stenting have significantly reduced the rate of major urological complications after kidney transplantation.^1^ Ureteral stenosis represents up to 50% of all urological complications after kidney transplantation and may affect 2% to 7.5% of patients.^2,3^ Inguinal hernia is a common complication of peritoneal dialysis, affecting up to 37% of patients, and may present rarely in the post-transplant follow-up of kidney transplant recipients.^4–6^ In most cases, the clinical course of inguinal hernia repair is uneventful, but inguinal herniation of a transplanted ureter may, in rare instances, cause obstructive uropathy with graft dysfunction in kidney transplant recipients.^7–19^

We report a unique case of acute renal failure due to ureteral obstruction after a mesh-plug inguinal hernia repair in a kidney transplant recipient. The patient involved in this study gave his written informed consent authorizing all procedures, and ethical consent for this study was not necessary as it did not involve any experimental treatment, and does not reveal any confidential information about patient that could violate their privacy

We also review the clinical features of urological complications caused by inguinal hernia surgery.

## CASE REPORT

A 62-year-old man with an end-stage renal disease secondary to polycystic kidney disease underwent successful deceased donor kidney transplantation. Six years after transplantation, the patient presented with vague abdominal pain and an inguinal hernia. His renal function was stable (SCr 1.5 mg/dL), and preoperative abdominal ultrasonography demonstrated a well-functioning kidney with no hydronephrosis and the presence of a direct inguinal hernia. An elective, open mesh-plug repair of the inguinal hernia was performed, by opening the external oblique muscle in the direction of the muscle fibers and reinserting the direct sac into the abdominal cavity, without opening the sac. We subsequently inserted an umbrella-shaped plug into the posterior wall defect and secured it with 3 interrupted Prolene^®^ stitches. We placed an unsecured onlay mesh patch with the ends draped around the cord structures at the level of the internal ring. We did not note the donor transplanted ureter and bladder during dissection, neither medial nor superior to the right-sided inguinal hernia, and we completed the surgical intervention without intraoperative complications.

**FIGURE 1:**
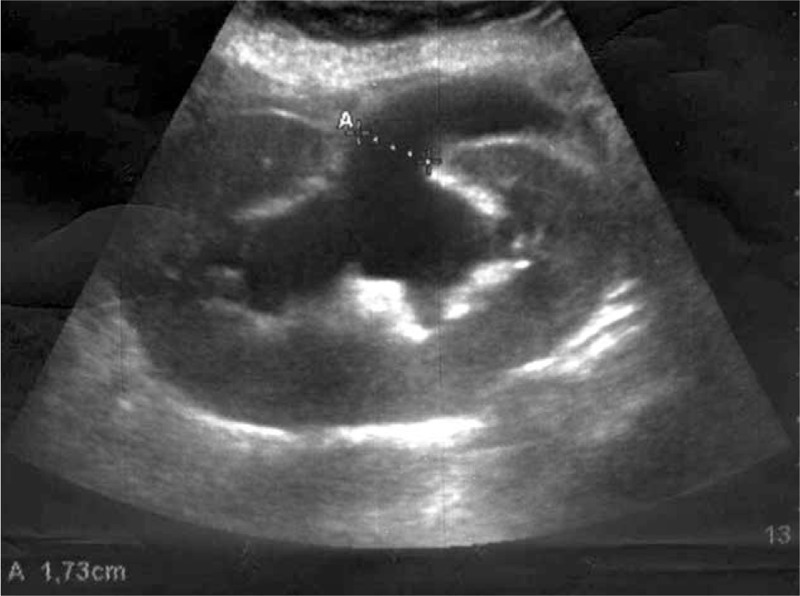
Graft sonography performed 6 h after hernia repair demonstrates severe hydronephrosis.

**FIGURE 2:**
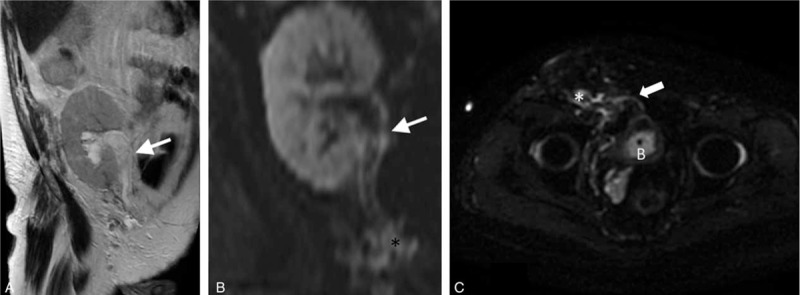
Magnetic resonance imaging performed 2 days after reoperation demonstrates mild, residual hydronephrosis and minor fluid collection (∗) at the site of previous plug positioning, near the passage of the ureter (arrow). B: bladder.

**FIGURE 3:**
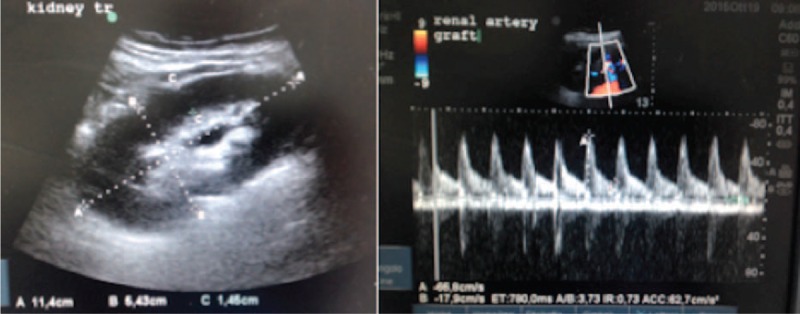
Graft sonography performed on the fourth postoperative day demonstrates near-complete resolution of hydronephrosis with a normal pulsatility index.

Immediately after the surgery, the patient became oliguric and then anuric. A graft sonography performed 6 hours after the hernia repair revealed massive hydroureteronephrosis (Figure 1). The distal portion of the ureter appeared to be absent. The serum creatinine level increased rapidly to 4.1 mg/dL; we decided to reoperate on the patient, still anuric, 8 hours after the first surgical procedure. During surgery, we did not identify the ureter, but there was a progressive increase in urine output immediately after plug removal. The transversalis fascia defect was closed, therefore, by the 3 interrupted Prolene^®^ stitches without plug interposition. We then placed an onlay mesh and completed the hernia repair using the standard technique. The postoperative course was uneventful and there was a progressive improvement in graft function. Three days after reoperation, the serum creatinine level decreased to 1.5 mg/dL and graft sonography demonstrated significantly reduced hydronephrosis. Magnetic resonance imaging (MRI) of the lower abdomen, performed 2 days after surgery, demonstrated fluid collection in the inguinal region at the site of previous plug implantation, near the passage of the ureter, confirming that the plug was responsible for ureteral compression (Figure 2). The patient was discharged 4 days after surgery, in good health, with stable renal function, and graft sonography demonstrated near-complete resolution of hydronephrosis (Figure 3).

## DISCUSSION

To our knowledge, this is the first reported case of acute renal failure in a kidney transplant recipient after inguinal hernia mesh-plug repair. Inguinal hernia repair is one of the most common surgical interventions performed worldwide. By some estimates, ∼0.8% of the general population in developed countries undergoes surgical repair in a 5-year period.^20^ Based on the European Hernia Society 2014 update of the Guidelines for the Treatment of Inguinal Hernia, all adult men with symptomatic inguinal hernia should be treated using a mesh procedure, regardless of the hernia type.^21–23^ Open Lichtenstein and mesh-plug techniques, as well as trans-abdominal preperitoneal (TAPP) and totally extraperitoneal (TEP) endoscopic techniques, are currently the methods of choice for the treatment of primary unilateral inguinal hernia. Open Lichtenstein and mesh-plug techniques provide similar outcomes in terms of hernia recurrence and chronic pain,^21–23^ although recent evidence suggests a lower likelihood of reoperation for mesh-plug repair.^24^ Compared with the Lichtenstein repair, endoscopic TEP and TAPP repairs have the advantages of lower rates of wound infection as well as earlier resumption of normal working activity, and, most importantly, a lower risk of chronic pain.^25,26^

An increased risk of urological complications was reported recently in patients with a previous prosthetic hernia repair undergoing kidney transplantation. Nadalin et al^27^ described a kidney transplant recipient who underwent laparoscopic TAPP repair of a symptomatic bilateral inguinal hernia 2 years earlier: at surgery, the fundus of the bladder and the pubis were completely encased in fibrous tissue compounded with mesh, making the extraperitoneal dissection of the bladder exceptionally challenging. More recently, Tse and Clancy^10^ reported a patient who developed stenosis of the renal transplant ureter after a laparoscopic repair of a bilateral recurrent inguinal hernia. The transplanted ureter was obstructed and adhered to the mesh. After dissection from the mesh, the stenotic segment was resected and the ureter was reanastomosed over a ureteric stent to the bladder. Similar findings were encountered in patients undergoing an open mesh-plug repair before transplantation. Weale et al^28^ reported surgical technical difficulties in 4 patients who underwent kidney transplantation on the same side of previous mesh-plug hernia repairs because of adherence of surrounding structures to the mesh and fixation of the cord. Ortiz et al^29^ reported ureteral necrosis in a kidney transplant recipient with a previous mesh-plug hernia repair. In this patient, the ureteral necrosis may have been related to an inflammatory reaction near the transplanted ureter, which was proximal to the implanted prosthetic mesh. Taken together, these studies suggest that kidney transplantation should be performed in the contralateral site of a previously implanted, unilateral mesh for inguinal hernia repair, and, in cases of bilateral hernia, laparoscopic repair with mesh should be avoided whenever possible.^10,27–29^

Inguinal hernia after renal transplantation may present as a urological complication because of herniation into the inguinal canal of the transplanted ureter. A total of 13 cases of inguinal herniation of a transplanted ureter have been reported in the literature (Table 1).^7–19^ Factors that may contribute to inguinal herniation of the transplant kidney include the existence of a redundant long ureter, placement of donor ureter over the spermatic cord, obesity, and stricture of the ureterovesicular anastomosis leading to twisting and kinking of the ureter into the inguinal canal.^7,11,17,19^ Older male patients, with a median age of 56.9 years, were more commonly affected. Inguinal herniation of the ureter is a late cause of obstructive uropathy and presented at a mean of 10.6 years (range 2–20, years) after transplantation. Almost all patients presented with graft dysfunction together with inguinal pain. Graft sonography usually demonstrated various degrees of hydronephrosis, and, in most cases, the diagnosis was confirmed by computed tomography or MRI, which allowed a clear picture for surgical planning. Although conservative treatment has been described,^9^ most patients required a nephrostomy procedure before elective surgical repair. The definitive treatment was inguinal herniorrhaphy without the need for ureter reimplantation. However, in the case of ureter necrosis,^12^ long redundant ureter^8,17^ or stricture of the ureter,^10^ a ureteral resection with reanastomosis, was required.

**TABLE 1:**
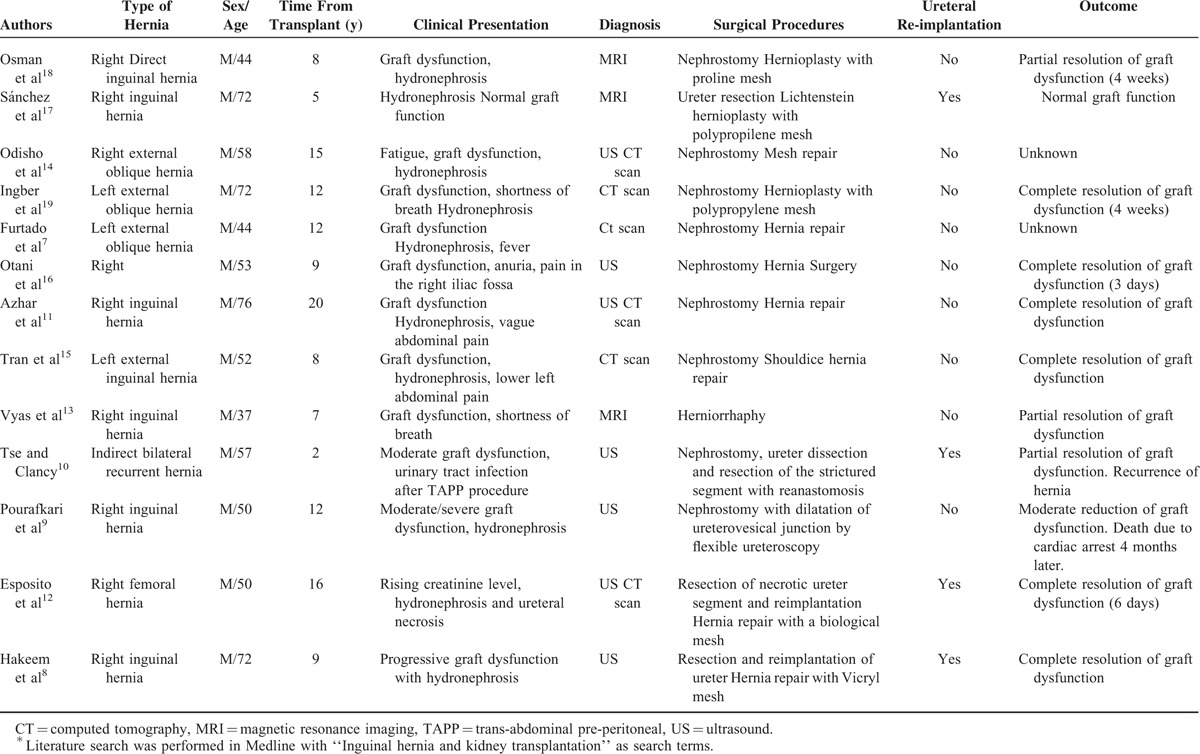
Analysis of Reported Cases of Obstructive Uropathy Caused by Inguinal Hernia in Kidney Transplant Recipients^∗^

**FIGURE 4:**
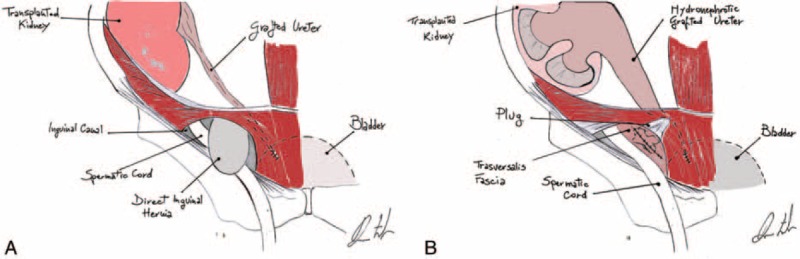
Mechanism of ureteral compression. (A) The patient presents with a direct inguinal hernia, without herniation of the transplanted ureter. (B) The plug is inserted into the preperitoneal space to reduce the direct hernia and causes direct compression of the ureter and its complete occlusion.

Acute renal failure after inguinal hernia repair is reported rarely among kidney transplant recipients. Selman et al^30^ reported a case of anuria in a renal transplant recipient following inguinal herniorrhaphy due to accidental ligation of the ureter, and successful treatment included a nephrostomy and ureteral reimplantation. In the present case, we attributed acute renal failure to ureteral compression by the plug that was placed in the preperitoneal space (Figure 4). Immediate surgical reintervention with plug removal resulted in rapid improvement of graft function, and the patient recovered completely 4 days after reintervention.

In conclusion, although mesh-plug inguinal hernia repair is a safe surgical technique, this unique report highlights that kidney transplant recipients presenting inguinal hernia may be predisposed to a higher risk of urological complications and graft dysfunction. Surgeons must be aware of graft and ureter positions before proceeding with hernia repair. A prompt diagnosis with graft sonography and abdominal computed tomography scan, followed by emergency surgery, may avoid the need for nephrostomy positioning and may improve the resolution of graft dysfunction.
